# Proteomics Disclose the Potential of Gingival Crevicular Fluid (GCF) as a Source of Biomarkers for Severe Periodontitis

**DOI:** 10.3390/ma15062161

**Published:** 2022-03-15

**Authors:** Elisa Bellei, Carlo Bertoldi, Emanuela Monari, Stefania Bergamini

**Affiliations:** 1 Proteomic Lab, Department of Surgery, Medicine, Dentistry and Morphological Sciences with Transplant Surgery, Oncology and Regenerative Medicine Relevance, University-Hospital of Modena and Reggio Emilia, Via del Pozzo 71, 41124 Modena, Italy; elisa.bellei@unimore.it (E.B.); emanuela.monari@unimore.it (E.M.); 2 Unit of Dentistry and Oral-Maxillofacial Surgery, Periodontology Section, Department of Surgery, Medicine, Dentistry and Morphological Sciences with Transplant Surgery, Oncology and Regenerative Medicine Relevance, University-Hospital of Modena and Reggio Emilia, Via del Pozzo 71, 41124 Modena, Italy; carlo.bertoldi@unimore.it

**Keywords:** gingival crevicular fluid, biomarkers, periodontitis, periodontal disease, proteomics, SDS-PAGE, mass spectrometry

## Abstract

Periodontal disease is a widespread disorder comprising gingivitis, a mild early gum inflammation, and periodontitis, a more severe multifactorial inflammatory disease that, if left untreated, can lead to the gradual destruction of the tooth-supporting apparatus. To date, effective etiopathogenetic models fully explaining the clinical features of periodontal disease are not available. Obviously, a better understanding of periodontal disease could facilitate its diagnosis and improve its treatment. The purpose of this study was to employ a proteomic approach to analyze the gingival crevicular fluid (GCF) of patients with severe periodontitis, in search of potential biomarkers. GCF samples, collected from both periodontally healthy sites (H-GCF) and the periodontal pocket (D-GCF), were subjected to a comparison analysis using sodium dodecyl sulphate-polyacrylamide gel electrophoresis (SDS-PAGE). A total of 26 significantly different proteins, 14 up-regulated and 12 down-regulated in D-GCF vs. H-GCF, were identified by liquid chromatography-tandem mass spectrometry (LC-MS/MS). The main expressed proteins were inflammatory molecules, immune responders, and host enzymes. Most of these proteins were functionally connected using the STRING analysis database. Once validated in a large scale-study, these proteins could represent a cluster of promising biomarkers capable of making a valuable contribution for a better assessment of periodontitis.

## 1. Introduction

Periodontal diseases (PDs) are oral infection diseases characterized by gingival inflammation, clinical attachment loss, and alveolar bone resorption. PDs are very common and can impact on 90% of the worldwide population [1], while approximately 10–15% of individuals are affected by a severe form of periodontitis [2]. Specifically, periodontitis is a chronic immune-inflammatory disease associated with a dysbiotic biofilm and characterized by the progressive destruction of the periodontium. The disease evolves in different ways and with differing progression. However, PDs result in periodontal attachment loss, which increases over time [3]. Because of their potential severity, more in-depth research is needed, including the development of reliable disease biomarkers [1]. Proteomics represents an emerging area of interest in the study of oral health and disease conditions, including periodontitis, gingivitis, dental caries, and oral cancer [4]. Moreover, it has an important role in molecular medicine, as it provides the linkage among genes, proteins, and disease, so it could have promising relevance in the field of periodontal diagnostics [5]. Particularly, periodontal proteomics can be applied to periodontology to develop diagnostic tools for the early detection of the disease onset, as well as to improve treatments and monitor outcomes [6]. Human gingival crevicular fluid (GCF) is a complex mixture defined as the transudate of the gingival tissue interstitial fluid produced physiologically by an osmotic gradient. During inflammatory challenges, in relation to the depth of the periodontal pocket, GCF becomes an inflammatory exudate containing a great number of proteins and peptides deriving from host inflammatory cells, oral bacteria, and structural cells of the periodontium and periodontal pocket [7]. Therefore, the analysis of the GCF constituents can reflect the disease status, allowing the identification of novel combinations of biomarkers capable of predicting periodontal health or disease [8]. The practical technical advantages of GCF are its easy, rapid, safe, painless, and non-invasive collection, without causing discomfort to the patient; moreover, its abundant cellular and molecular components can currently be analyzed with high accuracy through discovery methodologies, such as proteomics [9,10]. Historically, the origin and function of GCF has been controversial, mainly concerning whether it results from a physiological or pathological process [11]. Currently, GCF is recognized as a human body fluid approved as biological sample for the diagnosis and maintenance of a disease state [9]. A recent study shows that the cytomorphometric analysis of GCF allows for the quantitative evaluation of the inflammation dynamics in the periodontium for the identification of etiological risk factors of PDs following the use of fixed dental prostheses [12]. Furthermore, the comparative assessment of the levels of some cytokines in GCF from periodontally healthy subjects and patients with periodontitis and gingivitis can help to define the clinical status of the diseased site in chronic periodontitis [13].

Periodontal surgery is a basic element of active therapy and forms phase 2 of periodontal treatment, allowing for successful care of periodontal defects that would be unlikely through only the cause-related therapies. Thus, it is reasonable that periodontal defects that require periodontal surgery show more prominent features of the disease than defects that heal with causal therapy alone.

The aim of this study was to analyze and compare periodontally healthy and diseased GCF samples obtained from patients with severe periodontitis in order to identify differentially expressed proteins as potential diagnostic and prognostic biomarkers. The proteomic analysis was performed by sodium dodecyl sulphate-polyacrylamide gel electrophoresis (SDS-PAGE) associated with liquid chromatography-tandem mass spectrometry (LC-MS/MS). All the identified proteins were then subjected to a STRING analysis database to explore the feasibility of a functional association network among the differential proteins.

## 2. Materials and Methods

### 2.1. Reagents, Chemicals, and Solvents

Protein assay dye reagent, 2× Laemmli Sample Buffer and Precision Plus Protein™ Standards Dual Blue and Coomassie Blue G-250 were purchased from Bio-Rad Laboratories (Milan, Italy). Phosphate-buffer saline, bovine serum albumin, 2-mercaptoethanol, urea, thiourea, dithiothreitol, iodoacetamide, ammonium bicarbonate, trifluoroacetic acid, 3-[(3-Cholamidopropyl)-dimethylammonio]-propane-sulfonate (CHAPS), of molecular biology grade and high purity, were obtained from Merck KGaA (Milan, Italy). Protease inhibitors were furnished by Roche Italia, (Monza, MB, Italy). Solvents of MS purity grade, i.e., acetone, acetonitrile, methanol, formic acid, and glacial acetic acid were obtained from Carlo Erba Reagents Srl, (Milan, Italy). Precast gel Bolt™ 12% and 4–12% Bis-Tris Plus, and MES SDS-PAGE Running Buffer 10X, were purchased from Life Technologies Italia (Monza, MB, Italy). Trypsin Gold, Mass Spectrometry Grade, was obtained from Promega Corporation (Milan, Italy). Versylene sterile water (Fresenius Kabi, Isola della Scala, Italy) was used for gel washes and rinses and to prepare the solutions used in the described protocols.

### 2.2. Patient Selection

This study was performed at the Unit of Dentistry and Oral-Maxillofacial Surgery, Periodontology Department, of the University Hospital of Modena, Italy, after the approval of the local Ethics Committee of Health Services of the Emilia-Romagna Region (protocol n. 3968/2017, registration n. 315/2017), in accordance with the principles of the Helsinki Declaration (last edition 2013). All subjects were informed about the protocol and the aim of the study and signed an informed consent before their enrollment. The periodontal condition was carefully evaluated and treated with cause-related therapy when needed. Emergencies, such as pain or acute dental-periodontal infections, were also treated to establish a stable dental-periodontal condition. The periodontal assessment was carried out according to the criteria established by the World Workshop on the Classification of Periodontal and Peri-Implant Diseases and Conditions [14]. Patients with a diagnosis of severe periodontitis stage III or IV [14,15] and requiring periodontal surgery were screened [14,15,16,17,18]. At screening, the inclusion criteria were age (from 18 to 70 years), non-pregnant or lactating, non-smokers and without history of alcohol abuse, a medical history of good health, and the absence of bone diseases. Exclusion criteria were anti-inflammatory drugs consumption, uncontrolled or poorly controlled diabetes, unstable or life-threatening conditions, or requiring antibiotic prophylaxis [19,20]. At the conclusion of cause-related therapy, the inclusion criteria were a full-mouth plaque score (FMPS) ≤ 15%, full-mouth bleeding score (FMBS) ≤ 15%, and high levels of compliance [21,22]. Based on the above, 12 subjects (6 male and 6 female) without relevant health disorders were enrolled in the study (ages ranged from 40 to 70 years; mean 52.6 years ± 8.4, SD).

### 2.3. GCF Samples Collection

GCF samples were collected by the same trained clinician (C.B.) six weeks after the conclusion of the cause-related therapy (at baseline) and immediately before the periodontal surgery, following an intrasulcular absorption method [23,24]. Prior to GCF collection, the intervention site was isolated and dried with cotton rolls. Moreover, to prevent contamination, saliva was removed from the crevicular sulcus of the designated tooth by aspiration. This was a split-mouth designed study; small strips of sterile filter paper (Whatman 67 gr/m^2^, TISCH Scientific, Cleves, OH, USA) were positioned at 1–2 mm depth in the gingival crevice correspondent to healthy teeth, without evident signs of gingivitis or periodontitis before the start of causative therapy (H-GCF). The same GCF harvesting procedure was performed at the deeper periodontal pockets (the surgical defects) in the same patients (D-GCF). Therefore, the eventual differences in the GCF proteome between sites requiring periodontal surgery in addition to cause-related therapy (D-GCF) and sites not affected by any disease (H-GCF) should be easier to pinpoint. Overall, 12 H-GCF samples were taken from clinically healthy teeth and an equal number from teeth with severe periodontitis (D-GCF). The paper strips were left to absorb GCF for 30 s and were then removed; the strips from the same site were placed in the same sealed sterile tubes (Eppendorf Italia Srl, Milan, Italy; 1.5 mL volume). Bleeding on probing could not be detectable at teeth considered for GCF harvesting, so the strips visibly contaminated with blood were discarded and not considered for the study, while the others were immediately stored at −80 °C to minimize possible protein degradation and to avoid data misinterpretation [25].

### 2.4. GCF Protein Extraction and Quantification

After thawing, the paper strips were covered with 200 μL of phosphate buffer saline (PBS) 1× and incubated at +4 °C for 2 h in constant agitation. Subsequently, the strips were transferred into a new collection tube and centrifuged at 5000× *g* for 5 min at +4 °C to recover all the GCF content; the supernatant was then added to the eluate obtained after PBS incubation. Proteins extracted from GCF samples were precipitated overnight with cold acetone (1:12, *v*/*v*) at –20 °C. Finally, samples were centrifuged at 5000× *g* for 15 min at +4 °C; the protein pellet was resuspended in 60 μL of rehydration buffer (6 M urea, 2 M thiourea, 4% CHAPS, 25 mM dithiothreitol, protease inhibitors) and vortexed to obtain a finely solubilized sample. The H-GCF samples were mixed to form three pools (4 GCF samples per pool); the same was done for the D-GCF samples.

The total protein quantification of each pool was performed using the Bradford assay method, for 3 times on different days. Each sample (2 μL) was analyzed in duplicate, using the protein assay dye reagent (1:5 dilution) as the colorimetric component. A six-point standard curve (ranging from 0.2 to 6 μg/μL) was obtained with bovine serum albumin as the calibrator. The spectrophotometric reading was carried out in a microplate reader (Multiskan^TM^ Fc, Thermo Fisher Scientific, Waltham, MA, USA) at a wavelength of 595 nm.

### 2.5. SDS-PAGE Separation

The GCF protein extracts were subjected to SDS-PAGE analysis under reducing conditions. An aliquot of each pool (8 µg of protein content) was combined with 2× Laemmli sample buffer (62.5 mM Tris-HCl, pH 6.8, 25% glycerol, 2% SDS, 0.01% bromophenol blue) including 0.5% 2-mercaptoethanol. Protein denaturation was obtained by heating the mixture at +95 °C for 5 min in a Thermomixer Comfort device (Eppendorf, Milan, Italy). Protein separation was then carried out using precast gel Bolt^TM^ 12% Bis-Tris Plus and 4–12% Bis-Tris Plus, 12 wells. The electrophoretic run was performed in a Mini Protean Tetra Cell device (Bio-Rad, Hercules, CA, USA) using MES SDS 1× as the running buffer, at 100 V for 30 min, and next at 200 V for the remaining run time. After rinsing with pure water, the gels were stained overnight at constant moderate shaking with Coomassie Blue G-250, and de-stained with 30% methanol/10% glacial acetic acid solution. The GS-800 Calibrated Imaging Densitometer (Bio-Rad, Hercules, CA, USA) was used to acquire the gel images.

### 2.6. LC-MS/MS Analysis

The differentially expressed bands between the H-GCF and D-GCF samples were cut from the gels and were subjected to an “in-gel” trypsin digestion protocol, as previously described [26]. Briefly, gel bands were first de-stained with acetonitrile/ammonium bicarbonate, then reduced by 2.5% dithiothreitol at +56 °C and alkylated with 1% iodoacetamide. Proteins were digested with Trypsin Protease, MS grade (12 ng/mL) at +37 °C; the peptides were extracted with acetonitrile/trifluoroacetic acid and concentrated in a Concentrator Plus (Eppendorf Italia Srl, Milan, Italy). Prior to MS analysis, the dry samples were resuspended in 40 μL of acetonitrile/formic acid solution, sonicated for 10 min and centrifuged. Protein identifications were achieved using an UHPLC Q-Exactive MS (Thermo Fisher Scientific, Reinach, Switzerland), composed of a UltiMate 3000 HPLC System connected to an Q-Exactive Hybrid Quadrupole-Orbitrap™ mass spectrometer (LC-ESI-QO-MS/MS System); the analyses were performed as previously reported in detail [27].

### 2.7. Data Processing and Statistics

The Quantity One 1-D image analysis software (version 4.6.7, Bio-Rad) was used to detect the differentially expressed protein bands between healthy and diseased GCF pools. This software detects the protein signal intensity as optical density for each band, allowing the discovery of differential protein bands in different samples. A normalization procedure of the bands’ volumes was set to correct the variability that can occur during the gel staining process. Furthermore, a background image subtraction was applied to minimize noise density and background while maintaining the data integrity. An expression change of >1.5-fold revealed a significant difference between the H-GCF and D-GCF protein bands. After LC-ESI-QO-MS/MS analysis, the MS data were examined using the Mascot search engine (www.matrixscience.com, accessed on 9 May 2021) against both databases, neXtProt (for peptide sequences) and cRAP (for contaminants), setting the following parameters: Human taxonomy, trypsin as the proteolytic enzyme (one missed cleavage permitted), Cys carbamidomethylation (fixed modifications), Met oxidation plus Asn and Gln deamidation (variable modifications), mass tolerance 10 ppm and 0.05 Da (for precursor and product ions, respectively), and false discovery rate < 1%. Protein identification was performed, in triplicate, from bands excised from replicated lanes of H-GCF and D-GCF pools. The identified proteins were further investigated using STRING, a biological database of known and predicted protein-protein interactions containing information from various sources, such as experimental data, computational methods, and public scientific articles (online version 11.5; string-db.org, accessed on 25 October 2021). The analysis settings were as follows: full STRING network type, meaning of network edges (evidence), all the active interaction sources (text mining, experiments, co-expression, gene fusion, co-occurrence, neighborhood), and medium confidence as the minimum required interaction score (0.400). The Student’s t-test was employed to analyze data between healthy and diseased GCF samples, considering the differences as statistically significant when *p*-values were <0.05.

## 3. Results

### 3.1. SDS-PAGE

**Figure 1:**
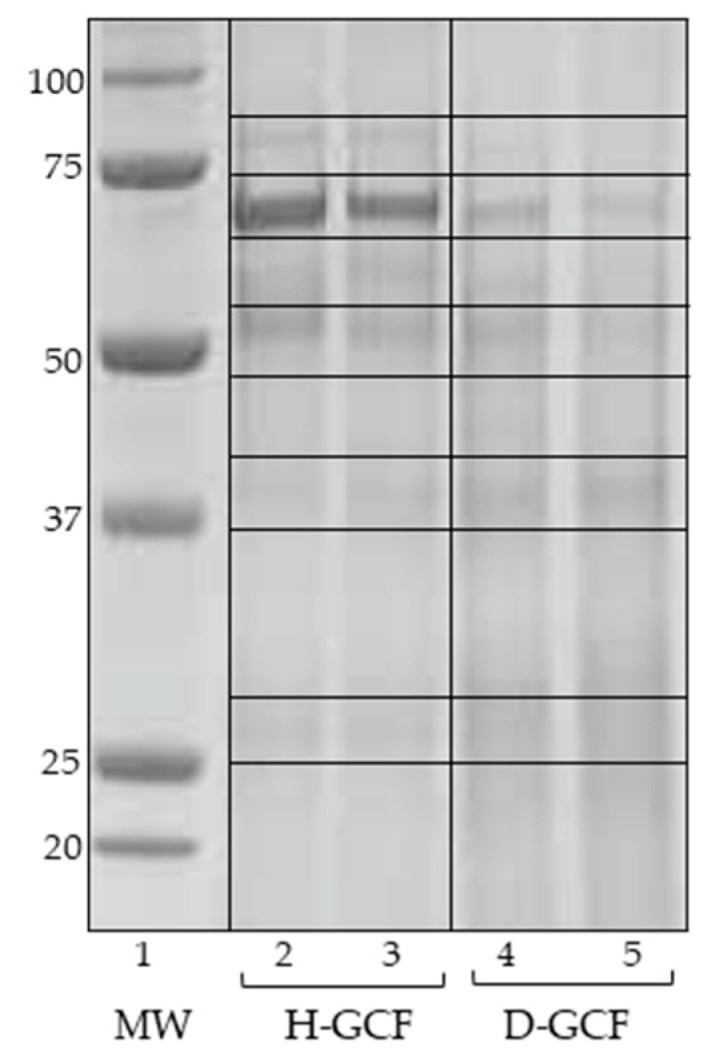
The representative SDS-PAGE image. First lane, MW: molecular weight marker (kDa), Dual Blue Precision Plus Protein Standard. Lanes 2–3, H-GCF: representative pools of H-GCF samples. Lanes 4–5, D-GCF: representative pools of D-GCF samples. The differentially expressed bands are enclosed within lanes. Gel separation: gradient Bolt 4–12% Bis-Tris Plus, Coomassie Blue stain method.

The proteins extracted from the GCF samples were separated by SDS-PAGE analysis. The separation profiles are shown in Figure 1.

The first lane of the gel image reports the molecular weight protein standard (Precision Plus Protein™ Standards Dual Blue), while lanes 2–3 and 4–5 refer to representative pools of healthy and pathological GCF samples, respectively. As clearly evidenced by the Coomassie Blue stain method, the two samples showed a distinctive and different proteomic profile. Only the bands with an expression difference >1.5-fold, detected by the Quantity One software, were selected for protein identification. These significant bands are enclosed between the lines (Figure 1), and the identified proteins are listed in Table 1.

To assess the best protein separation profile, we employed precast gels at different acrylamide concentrations, that is precast gel Bolt^TM^ 12% Bis-Tris Plus and precast gel Bolt^TM^ 4–12% Bis-Tris Plus; the gradient gels provided the finest bands resolution and separation. Moreover, the gels were comprised of 15 wells, thus allowing the simultaneous analysis of all GCF samples in the same electrophoretic run, avoiding the probability of differences arising among the analysis performed in different experiments.

### 3.2. LC-MS/MS Identifications

Proteins in the bands were identified by LC-ESI-QO-MS/MS analysis. All the differential proteins are listed in Table 1 as up- or down-regulated proteins in D-GCF.

The protein identification searches were limited to the human protein sequence database. The first and second column of Table 1 refer to the primary protein full name and to the relative gene name, respectively. In column three, the accession number from the neXtProt database is reported, while the subsequent columns show the mass data: the highest score derived from the Mascot search engine (accessed on 9 May 2021), the experimental mass, and the exponentially modified Protein Abundance Index (emPAI), that estimate the absolute protein contents in complex mixture (number of observed peptides/number of observable peptide per protein) [28]. Some deviations between the molecular weights in the gel and the monoisotopic mass derived from MS analysis could be due to post-translational modifications. The values of significant peptides and significant matches were ≥ 2, and the percentage of sequence coverage ranged from 4 to 48%. The last column provides the fold-change of the protein signal, calculated by densitometric analysis as the ratio between the OD intensity of D-GCF vs. H-GCF. A total of 26 proteins (14 up-regulated and 12 down-regulated in periodontal-related samples) showed a 1.5-fold difference in expression between healthy and diseased (*p* < 0.05) samples. The greatest increases were observed for A2M (fold-change +3.00), HP (fold-change +2.66), IGHG1 and IGHG2 (fold-change +2.33 and +2.29, respectively), and GAPDH (fold-change +2.00).

The main molecular and biological functions of the identified proteins are shown in Table 2, as basically deducted by the ExPASy database, a proteomic server for in-depth protein knowledge [29], UniProtKB, and neXtProt databases (accessed on 25 October 2021), as well as from the current available literature (PubMed.org) (accessed on 25 November 2021).

Most of the up-regulated proteins found in periodontal GCF principally resulted as related to inflammation (acute phase proteins) and to the immunity process (immunoglobulins). Among the down-regulated proteins, a prevalence of various isoforms of keratins (66%) was found, including an oral isoform. Some proteins were already identified in our previous searches conducted on periodontal pocket tissue [30,31] and tooth surface collected material (TSCM) [32]. Significantly, among all the identified proteins, we no traces of hemoglobin were identified, thus proving the optimal GCF collection and extraction protocols adopted in this study.

### 3.3. STRING Analysis

**Figure 2:**
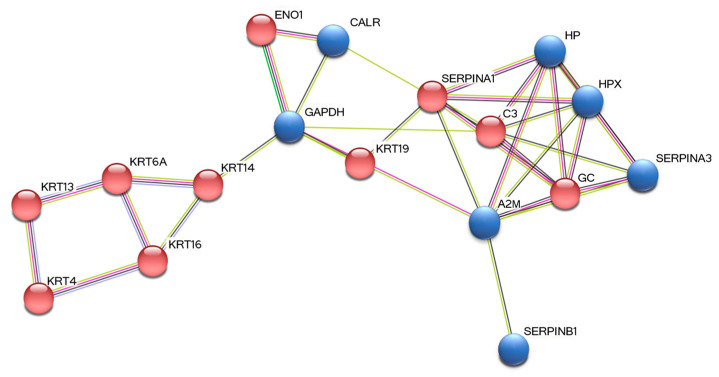
The functional interaction network obtained using STRING. Proteins are indicated with their gene names. Blue nodes = up-regulated proteins in D-GCF: CALR, calreticulin; GAPDH, glyceraldehyde-3-phosphate dehydrogenase; HP, haptoglobin; HPX, hemopexin; SERPINA3, alpha-1-antichymotrypsin; A2M, alpha-2-macroglobulin; SERPINB1, leukocyte elastase inhibitor. Red nodes = down-regulated proteins in D-GCF: ENO1, alpha-enolase; SERPINA1, alpha-1-antitrypsin; KRT19, keratin, type I cytoskeletal 19; C3, complement C3; GC, Vitamin-D-binding protein; KRT14, keratin, type I cytoskeletal 14; KRT16, keratin, type I cytoskeletal 16; KRT6A, keratin, type II cytoskeletal 6A; KRT13, keratin, type I cytoskeletal 13; KRT4, keratin, type II cytoskeletal 4.

The analysis of the identified proteins using the STRING database allowed us to create an interaction pathway map between the differential proteins found in GCF (Figure 2). Specifically, 17 of the 26 discovered proteins resulted as functionally related to each other (average node degree = 3.18). Seven were up-regulated proteins, indicated with blue nodes (A2M, SERPINB1, SERPINA3, HP, HPX, GAPDH, CALR) and 10 were down-regulated proteins, showed by red nodes (KRT13, KRT14, KRT16, KRT19, KRT4, KRT6A, ENO1, GC, C3, SERPINA1). Only five proteins were disconnected (EIF4G3, TMEM201, SPTLC3, KRT1, KRT76) and four proteins were not detected by the database (IGHG1, IGHG2, IGHA1, IGHV3-74). All proteins are indicated with the name of the relative gene (as reported in Table 1). Proteins associations are expressed by edges (n = 35; average local clustering coefficient = 0.38; PPI enrichment *p*-value < 1.0 × 10^−16^). Direct known interactions are displayed by pink and blue lines, while predicted connections are expressed by green and black lines.

## 4. Discussion

Periodontal disease is characterized by inflammation and clinical attachment loss [33]. Microbiota and immuno-inflammatory pathways have been involved in the study of periodontal disease to build up new theoretical models of the pathogenesis of periodontitis, which include the microbiota activation of immuno-inflammatory mechanisms. Generally, the oral disorders, including periodontitis, are multifactorial polygenic diseases whose etiology is attributed to numerous different genes and the encoded proteins; accordingly, their pathophysiology may involve several biological pathways [34]. However, these pathogenetic patterns did not define the dynamic nature of the involved biochemical processes. A modern pathogenetic conceptualization incorporating gene, protein, and metabolite data into biologic processes based on a multilevel framework that include disease-initiating and -resolving mechanisms is required. From this point of view, the identification of candidate proteins using proteomic tools, together with the molecular connections among proteins, may allow for the establishment of a proper framework of interactions, revealing potential dentistry biomarkers [35].

In this study, by a proteomic approach, we detected a cluster of differentially expressed proteins between healthy and periodontally diseased GCF samples (Figure 1 and Table 1). Then, using the STRING database, we generated a functional protein network composed of seven of the up-regulated proteins (A2M, SERPINB1, SERPINA3, HP, HPX, GAPDH, CALR,) and 10 of the down-regulated proteins (KRT13, KRT14, KRT16, KRT19, KRT4, KRT6A, ENO1, GC, C3, SERPINA1) (Figure 2). The principal functions of all the identified proteins are summarized in Table 2; the proteins already identified in our previous proteomic studies conducted on periodontal pocket tissue [30,31] and tooth surface collected material (TSCM) [32] are pointed out in this table.

Within the group of the up-regulated proteins, the most significant patterns were composed of proteins involved in inflammation (A2M, SERPINB1, SERPINA3, HP, and HPX) and in the immune response. A summary of the results can be viewed in Supplementary Materials (Figure S1). A2M is an acute-phase protein and a nonspecific proteinases inhibitor, including those involved in the chronic inflammatory process by protecting against both endogenous and exogenous inflammatory damage [36]. Moreover, A2M is considered an osteogenic growth peptide-binding protein capable of enhancing the availability of this peptide to its cellular targets and tissues [37]. Thus, the presence of high A2M levels in periodontal GCF could have a double implication, showing not only an anti-inflammatory function, but perhaps also acting to counter the tissue degradation and bone resorption processes present in periodontal disease. Notable, a recent study identified A2M among the top 20 GCF-abundant proteins [23]. SERPINB1 is a serine protease inhibitor with an essential role in the regulation of innate immune response, cellular homeostasis, and inflammation, limiting the activity of inflammatory caspases by suppressing their enzymatic activation [38]. It principally protects cells from proteases produced during stress or infection, reducing the destruction of host proteins and mortality. Reasonably, the increase of this protein in pathological GCF could be a protective response against the infectious and inflammatory condition present in PDs. In support of our findings, other previous works showed an up-regulation of this protein in the GCF of patients affected by chronic periodontitis in comparison to periodontally healthy subjects [39,40]. Like SERPINB1, SERPINA3 is also a serine proteinases inhibitor, whose concentration can increase about 4-fold during the inflammatory process [41]. It exerts a natural defense, as its main physiological target is neutrophil cathepsin G, an enzyme with a central role in the destruction of bacteria by neutrophils. Another inflammatory modulator found to be increased in the D-GCF samples is HP. This protein plays a fundamental role in many aspects of the acute phase response. Furthermore, it exhibits well-known and important antibacterial activity and a natural bacteriostatic function [42]. In agreement with our results, others proteomic studies evidenced an overexpression of HP in the GCF obtained from periodontal disease [43] and aggressive periodontitis [44]. Additionally, HPX, a glycoprotein belonging to the family of acute-phase reactants, whose synthesis is induced after an inflammatory event [45], was also found up-regulated in diseased GCF samples. HPX has an antioxidant activity, as it strongly binds heme at the extracellular level to inhibit its influx into the cells, thus conferring cytoprotection against intracellular toxicity and tissue damage [46]. Therefore, in the context of the present study, the increase of HPX could be considered a protective strategy as opposed to the oxidative stress condition and inflammation associated with severe periodontitis [47]. In the same way as A2M, SERPINB1, SERPINA3, and HP, as well as the high presence of HPX in D-GCF, could be interpreted as an attempt to modulate the high inflammatory state, as well as to counter the bacterial environment created with periodontal infection. Continued advances in dentistry have recognized the pivotal tasks of the host inflammatory and immune responses in periodontal disease progression, as well as how the host reaction in periodontitis is a complex interplay between various cell types, proteolytic enzymes, and inflammatory mediators [48]. In accordance, the evaluation of cytokines expression can offer valuable information into the mechanisms of initiation and advancement of PDs. In this regard, Papathanasiou et al. have reported increased levels of interferon-γ, but not of interleukin-4, interleukin-33, and thymic stromal lymphopoietin in GCF samples from inflamed sulcus of patients with chronic periodontal disease [13]. Other proteins found significantly overexpressed in our D-GCF samples comprise some immunoglobulins such as IGHG1, IGHG2, IGHA1, and IGHV3-74. Immunoglobulins are commonly present at the site of the periodontal injury, as they derive from both systemic and local tissue sources [44]. They act as an innate defense system of the periodontium through their interference with bacterial processes, and their occurrence is characteristic of the different stages and grades of periodontal disease [6]. The high presence of immunoglobulins in diseased GCF may be an active defense mechanism against the local pathogenic environment. Another protein found increased in D-GCF is GAPDH. This is a key enzyme in glycolysis; additionally, it plays a role in innate immunity by promoting TNF-induced NF-kappa-B activation and type I interferon production, as well as participating in nuclear events including transcription, RNA transport, DNA replication, and apoptosis (UniProtKB database). We previously revealed an overexpression of GAPDH in periodontal pocket tissue [31] and in TSCM after periodontal surgery and rinsing with chlorhexidine [32]. Moreover, Baliban et al. also reported the finding of bacterial GAPDH in GCF samples from chronic periodontitis [49]. Finally, we discovered an increase of peptides involved in protein biosynthesis, folding, and biosynthetic processes such as CALR, EIF4G3, TMEM201, and SPTLC3 (UniProtKB). Specifically, CALR promotes folding, oligomeric assembly, and quality control in the endoplasmic reticulum via the calreticulin/calnexin cycle. It is a molecular binding chaperone acting in the biosynthesis of myeloperoxidase, a lysosomal heme protein present in neutrophils and monocytes, necessary for efficient oxygen-dependent microbial activity [50]. We could assume that its high expression in the D-GCF samples might support the pathogenic bacteria present in the microbial biofilm and in gingival tissue during the development and progression of periodontal disease.

Twelve proteins resulted as down-regulated in D-GCF vs. H-GCF, 10 of which were strictly connected using STRING analysis. The largest group was composed of various forms of keratins. This is in accordance with the high presence of cytokeratins at the gingival level. The higher concentration detected in healthy samples compared to diseased ones may be due to the normal and very rapid turnover rate of the healthy sulcular epithelium [44]. Consequently, is not surprising that the H-GCF showed large amounts of keratins, probably derived from this dynamic turnover. On the other hand, the low presence of cytokeratins in periodontal disease can be attributed to the passive release of cellular debris from of the ulcerated pocket epithelium [51]. Other results obtained by similar proteomics research closely overlap with our findings, reporting all the eight types of keratins (KRT13, KRT14, KRT16, KRT19, KRT1, KRT4, KRT76, KRT6A) as decreased in GCF samples from severe chronic periodontitis [40,44]; KRT1 was also confirmed by Choi et al. [39]. KRT13, KRT19, and KRT4 were identified in our previous proteomic studies conducted on periodontal pocket tissue [31], as well as ENO1, a glycolytic enzyme involved in growth control and hypoxia tolerance [30,31]. Two other proteins were found to be down-regulated, namely GC, implicated in vitamin D transport and storage, and C3, a protein with a central role in the activation of the complement system (UniProtKB; neXtProt). In agreement, Bostanci et al. reported reduced amounts of both GC and C3 in GCF samples from aggressive periodontitis compared to healthy samples [44]. Finally, we showed a decrease of SERPINA1, an inhibitor of serine proteases and elastase.

In summary, some of the identified proteins confirm previous results, while others, to our knowledge, are new proteins revealed in GCF for the first time. This underlines the importance of these innovative findings, as they add significant novel insights into the molecular composition of GCF. However, it should be noted that, being a preliminary study, it was conducted on a small sample size. This is a noteworthy limitation; therefore, further experiments are needed to increase the number of cases for a large-scale validation before clinical use. Moreover, additional analysis using more advanced proteomic techniques (such as two-dimensional gel electrophoresis), as well as other databases of information on genes and their products (including Gene Ontology Resource), will help to better define these early findings. Markedly, the present results endorse the concept that it is unlikely to consider only a single molecule as a reliable biomarker for a complex oral pathology such as periodontitis [52]. Mass-scale genomics and proteomics outcomes become meaningful only if inserted in capillary networks of molecular interactions and protein-protein associations [34].

## 5. Conclusions

The GCF proteomic content between periodontal samples harvested from sites without the clinical features of periodontal disease (or of gingivitis) (H-GCF) and diseased samples (D-GCF) differs significantly. However, it is to consider that in this split-mouth designed study, all the patients suffered from periodontal disease. Therefore, the observed differences were between clinically affected and non-affected sites. Most of the up-regulated proteins detected in D-GCF were inflammatory proteins, immunoglobulins, and host enzymes, thus highlighting the nature of GCF as a local inflammatory exudate of the periodontal tissue. Furthermore, the high presence of host defense-related proteins contributes to defining the immune and inflammatory features of periodontitis. In conclusion, MS-based proteomics proved to be a suitable tool for a deeper understanding of oral diseases. Specifically, the identified proteins could represent a cluster of promising biomarkers for the management of PDs.

## Figures and Tables

**Table 1:** The differentially expressed human proteins between healthy and diseased GCF.

Protein Full Name ^a^	Gene ^b^	Acc. Number ^c^	Score ^d^	Mass ^e^	emPAI ^f^	Change ^g^
**Up-regulated proteins in D-GCF**						
Alpha-2-macroglobulin (fragment)	A2M	NX_P01023-1	18	164,613	0.05	+3.00
Leukocyte elastase inhibitor	SERPINB1	NX_30740-1	111	42,829	0.56	+1.75
Alpha-1-antichymotrypsin	SERPINA3	NX_01011-1	49	47,792	0.37	+1.94
Haptoglobin	HP	NX_P00738-1	42	45,861	0.09	+2.66
Hemopexin	HPX	NX_P02790-1	36	52,385	0.15	+1.86
Immunoglobulin heavy constant gamma 1	IGHG1	NX_P01857-1	143	36,596	0.86	+2.33
Immunoglobulin heavy constant gamma 2	IGHG2	NX_P01859-1	44	36,505	0.23	+2.29
Immunoglobulin heavy constant alpha 1	IGHA1	NX_P01876-1	15	38,486	0.10	+1.83
Immunoglobulin heavy variable 3-74	IGHV3-74	NX_A0A0B4J1X5-1	16	13,002	0.76	+1.67
Glyceraldehyde-3-phosphate dehydrogenase	GAPDH	NX_P044406-1	46	36,201	0.37	+2.00
Calreticulin	CALR	NX_P27797-1	21	48,283	0.08	+1.61
Eukaryotic translation initiation factor 4 gamma 3 (fragment)	EIF4G3	NX_O43432-1	24	177,682	0.02	+1.80
Transmembrane protein 201	TMEM201	NX_Q5SNT2-1	14	73,444	0.05	+1.80
Serine palmitoyltransferase 3	SPTLC3	NX_Q9NUV7-1	26	62,352	0.06	+1.75
**Down-regulated proteins in D-GCF**						
Keratin, type I cytoskeletal 13 isoform Iso 1	KRT13	NX_P13646-1	580	49,900	3.59	0.60
Keratin, type I cytoskeletal 14 isoform Iso 1	KRT14	NX_P02533-1	713	51,872	4.41	0.60
Keratin, type I cytoskeletal 16 isoform Iso 1	KRT16	NX_P08779-1	598	51,578	4.06	0.60
Keratin, type I cytoskeletal 19 isoform Iso 1	KRT19	NX_P08727-1	87	44,079	0.54	0.80
Keratin, type II cytoskeletal 1 isoform Iso 1	KRT1	NX_P04264-1	130	66,170	0.59	0.67
Keratin, type II cytoskeletal 4 isoform Iso 1	KRT4	NX_P19013-1	53	57,649	0.30	0.80
Keratin, type II cytoskeletal 2 oral isoform Iso 1	KRT76	NX_Q01546-1	150	66,370	0.78	0.43
Keratin, type II cytoskeletal 6A isoform Iso 1	KRT6A	NX_P02538-1	40	60,293	0.37	0.33
Alpha-enolase	ENO1	NX_P06733-1	108	47,481	0.62	0.80
Vitamin D-binding protein	GC	NX_P02774-1	28	54,480	0.15	0.66
Complement C3 (fragment)	C3	NX_P01024-1	205	188,569	0.30	0.55
Alpha-1-antitrypsin	SERPINA1	NX_P01009-1	76	46,878	0.50	0.50

^a^ Principal protein full name from neXtProt database; ^b^ Protein gene name; ^c^ Primary protein accession number from neXtProt database; ^d^ The highest score obtained with the Mascot search engine; ^e^ Monoisotopic mass (M_r_) from MS analysis (Da); ^f^ emPAI: exponentially modified protein abundance index; ^g^ Fold-change of protein expression between D-GCF and H-GCF.

**Table 2:** The main roles of the differentially expressed proteins in D-GCF with respect to H-GCF.

Prot. Abbr. ^a^	Principal Functions ^b^	Biological Process ^c^
**Up-regulated proteins**		
A2M	Calcium-dependent protein binding; endopeptidase inhibitor activity	Acute-phase response
SERPINB1	Protease inhibitor; regulation of immune response and inflammation	Inflammatory and immune response
SERPINA3	Protease inhibitor; acute-phase response	Inflammatory response
HP	Acute inflammatory response and defense response; antibacterial activity	Acute-phase response modulation
HPX	Acute-phase reactant; intracellular antioxidant; iron homeostasis	Acute-phase response
IGHG1	Antigen binding; immunoglobulin receptor binding	Immune response
IGHG2	Antigen binding; immunoglobulin receptor binding	Immune response
IGHA1	Antigen binding; antibacterial humoral response	Immune response
IGHV3-74	Participates in the antigen recognition and binding; immune response	Adaptive immunity
GAPDH (*,§)	Key enzyme in glycolysis; also plays a role in innate immunity	Energy metabolism
CALR	Calcium-binding chaperone; positive regulation of cell cycle	Protein folding
EIF4G3	Regulation of translational initiation; RNA binding	Protein biosynthesis
TMEM201	Actin filament and lamin binding; nuclear envelope organization	Transmembrane protein
SPTLC3	Acyltransferase; sphingolipid pathway; lipid metabolism	Biosynthetic processes
**Down-regulated proteins**		
KRT13 (*)	Structural molecule activity	Cytoskeleton organization
KRT14	Keratin filament binding; structural constituent of cytoskeleton	Epidermis development
KRT16	Epidermis-specific type I keratin; structural constituent of cytoskeleton	Cornification, keratinization
KRT19 (*)	Organization of myofibers; structural constituent of cytoskeleton	Cornification, keratinization
KRT1	Structural constituent	Fibrinolysis, keratinization
KRT4 (*)	Epithelial cell differentiation	Cornification, keratinization
KRT76	Contributes to terminal cornification	Keratinization
KRT6A	Structural constituent of cytoskeleton	Cornification
ENO1 (#,*)	Glycolytic enzyme; also involved in hypoxia tolerance	Glycolytic process
GC	Vitamin D binding, transport, and storage	Vitamin transport
C3	Central role in complement system activation	Complement activation
SERPINA1	Inhibitor of serine proteases and elastase	Enzymatic inhibitor

^a^ Abbreviations of protein names correspond to their gene name (see Table 1 for direct correspondence). ^b^ Principal functions and ^c^ biological processes are deducted by ExPASy, UniProtKB, neXtProt, and PubMed databases. Proteins identified in our previous studies in periodontal pocket tissue # [30] and * [31]; in tooth surface collected material (TSCM) § [32].

## Data Availability

All relevant data are included within the paper. The datasets used and/or analyzed during the current study are available from the corresponding author on reasonable request.

## Supplementary Materials

The following supporting information can be downloaded at: https://www.mdpi.com/article/10.3390/ma15062161/s1, Figure S1:The summary representation of the obtained results.

## Abbreviations

A2M	Alpha-2-macroglobulin
C3	Complement C3
CALR	Calreticulin
D-GCF	Diseased-Gingival Crevicular Fluid
EIF4G3	Eukaryotic translation initiation factor 4 gamma 3
ENO1	Alpha-enolase
GAPDH	Glyceraldehyde-3-phosphate dehydrogenase
GC	Vitamin D-binding protein
H-GCF	Healthy-Gingival Crevicular Fluid
HP	Haptoglobin
HPX	Hemopexin
IGHA1	Immunoglobulin heavy constant alpha 1
IGHG1	Immunoglobulin heavy constant gamma 1
IGHG2	Immunoglobulin heavy constant gamma 2
IGHV3-74	Immunoglobulin heavy variable 3-74
KRT1	Keratin, type II cytoskeletal 1 isoform Iso 1
KRT13	Keratin, type I cytoskeletal 13 isoform Iso 1
KRT14	Keratin, type I cytoskeletal 14 isoform Iso 1
KRT16	Keratin, type I cytoskeletal 16 isoform Iso 1
KRT19	Keratin, type I cytoskeletal 19 isoform Iso 1
KRT4	Keratin, type II cytoskeletal 4 isoform Iso 1
KRT6A	Keratin, type II cytoskeletal 6A isoform Iso 1
KRT76	Keratin, type II cytoskeletal 2 oral isoform Iso 1
LC-MS/MS	Liquid Chromatography/Tandem Mass Spectrometry
PBS	Phosphate Buffer Saline
PDs	Periodontal Diseases
SDS-PAGE	Sodium Dodecyl Sulphate-PolyAcrylamide Gel Electrophoresis
SERPINA1	Alpha-1-antitrypsin
SERPINA3	Alpha-1-antichymotrypsin
SERPINB1	Leukocyte elastase inhibitor
SPTLC3	Serine palmitoyltransferase 3
TMEM201	Transmembrane protein 201
TSCM	Tooth Surface Collected Material
