# Supplementary material for: Proteomics Disclose the Potential of Gingival Crevicular Fluid (GCF) as a Source of Biomarkers for Severe Periodontitis
Source: Materials (Basel). 2022 Mar 15;15(6):2161. doi: 10.3390/ma15062161 (PMC8950923; doi:10.3390/ma15062161)
Supplement: Supplementary file 1 [file materials-15-02161-s001.zip › materials-1557849-supplementary.pdf]

materials

MDPI

Article

Proteomics Disclose the Potential of Gingival Crevicular Fluid (GCF) as a Source of Biomarkers for Severe Periodontitis

Elisa Bellei [1], Carlo Bertoldi [2], Emanuela Monari [1] and Stefania Bergamini [1,*]

[1] Proteomic Lab, Department of Surgery, Medicine, Dentistry and Morphological Sciences with Transplant Surgery, Oncology and Regenerative Medicine Relevance, University-Hospital of Modena and Reggio Emilia, Via del Pozzo 71, 41124 Modena, Italy; elisa.bellei@unimore.it (E.B.); emanuela.monari@unimore.it (E.M.)

[2] Unit of Dentistry and Oral-Maxillofacial Surgery, Periodontology Section, Department of Surgery, Medicine, Dentistry and Morphological Sciences with Transplant Surgery, Oncology and Regenerative Medicine Relevance, University-Hospital of Modena and Reggio Emilia, Via del Pozzo 71, 41124 Modena, Italy; carlo.bertoldi@unimore.it

* Correspondence: stefania.bergamini@unimore.it; Tel.: +39-059-4223156

Abstract: Periodontal disease is a widespread disorder comprising gingivitis, a mild early gum inflammation, and periodontitis, a more severe multifactorial inflammatory disease that, if left untreated, can lead to the gradual destruction of the tooth-supporting apparatus. To date, effective etiopathogenetic models fully explaining the clinical features of periodontal disease are not available. Obviously, a better understanding of periodontal disease could facilitate its diagnosis and improve its treatment. The purpose of this study was to employ a proteomic approach to analyze the gingival crevicular fluid (GCF) of patients with severe periodontitis, in search of potential biomarkers. GCF samples, collected from both periodontally healthy sites (H-GCF) and the periodontal pocket (D-GCF), were subjected to a comparison analysis using sodium dodecyl sulphate-polyacrylamide gel electrophoresis (SDS-PAGE). A total of 26 significantly different proteins, 14 up-regulated and 12 down-regulated in D-GCF vs. H-GCF, were identified by liquid chromatography-tandem mass spectrometry (LC-MS/MS). The main expressed proteins were inflammatory molecules, immune responders, and host enzymes. Most of these proteins were functionally connected using the STRING analysis database. Once validated in a large scale-study, these proteins could represent a cluster of promising biomarkers capable of making a valuable contribution for a better assessment of periodontitis.

Keywords: gingival crevicular fluid; biomarkers; periodontitis; periodontal disease; proteomics; SDS-PAGE; mass spectrometry

Citation: Bellei, E.; Bertoldi, C.; Monari, E.; Bergamini, S. Proteomics Disclose the Potential of Gingival Crevicular Fluid (GCF) as a Source of Biomarkers for Severe Periodontitis. *Materials* **2022**, *15*, 2161. https://doi.org/10.3390/ma15062161

Academic Editors: Javier Gil, Georgios Romanos and Rafael Delgado-Ruiz

Received: 30 December 2021
Accepted: 11 March 2022
Published: 15 March 2022

Publisher's Note: MDPI stays neutral with regard to jurisdictional claims in published maps and institutional affiliations.

Copyright: © 2022 by the authors. Licensee MDPI, Basel, Switzerland. This article is an open access article distributed under the terms and conditions of the Creative Commons Attribution (CC BY) license (https://creativecommons.org/licenses/by/4.0/).

A total of 26 proteins were identified as differentially expressed: 14 were up-regulated and 12 were down-regulated in D-GCF compared to H-GCF. Most of the up-regulated proteins were involved in inflammation and immune response.

17 proteins resulted functionally associated by STRING analysis.

Up-regulated proteins

Inflammation	**A2M***
Inflammation	**SERPINB1***
Inflammation	**SERPINA3***
Inflammation	**HP***
Inflammation	**HPX***
Immunity	**IGHG1**
Immunity	**IGHG2**
Immunity	**IGHA1**
Immunity	**IGHV3-74**
Enzyme	**GAPDH***
Folding	**CALR***
Regulation	**EIF4G3**
Binding	**TMEM201**
Biosynthesis	**SPTLC3**

Maximum expression

Minimum expression

* Protein interaction (by STRING)

Down-regulated proteins

**KRT13***	Keratin
**KRT14***	Keratin
**KRT16***	Keratin
**KRT19***	Keratin
**KRT1**	Keratin
**KRT4***	Keratin
**KRT76**	Keratin
**KRT6A***	Keratin
**ENO1***	Enzyme
**GC***	Transport
**C3***	Activation
**SERPINA1***	Inhibition

Figure S1: Summary representation of the obtained results.
